# Cardiovascular Protection of Nephropathic Male Patients by Oral Food Supplements

**DOI:** 10.1155/2020/1807941

**Published:** 2020-06-13

**Authors:** Annalisa Noce, Giulia Marrone, Manuela Di Lauro, Silvia Urciuoli, Anna Pietroboni Zaitseva, Georgia Wilson Jones, Nicola Di Daniele, Annalisa Romani

**Affiliations:** ^1^UOC of Internal Medicine-Center of Hypertension and Nephrology Unit, Department of Systems Medicine, University of Rome Tor Vergata, Via Montpellier 1, 00133 Rome, Italy; ^2^School of Applied Medical, Surgical Sciences, University of Rome Tor Vergata, Via Montpellier 1, 00133 Rome, Italy; ^3^PHYTOLAB (Pharmaceutical, Cosmetic, Food Supplement, Technology and Analysis)-DiSIA, University of Florence, Via U. Schiff, 6, 50019 Sesto Fiorentino, Italy

## Abstract

Nephropathic patients show elevated cardiovascular morbidity and mortality compared to the general population. In order to delve deeper into the understanding of this phenomenon, it is necessary to recognize risk factors that are distinctive to the uremic state, such as oxidative stress and chronic low-grade inflammation. Moreover, gender differences have been reported in nephrology, as it has been observed that chronic kidney disease has higher prevalence in males than in females. The use of an oral food supplement (OFS) containing natural active compounds from *Capsicum annuum* L., Garcinia cambogia, *Centella asiatica* L., artichoke, and *Aesculus hippocastanum* L. which are virtually devoid from side effects, but rich in antioxidant and antiradical properties, could represent a valid therapeutic adjunct in the clinical management of nephropathic patients. Moreover, quantitative analysis performed *in vitro* on such compounds showed that they expressed good total antioxidant (7.28 gallic acid equivalents) and antiradical activity (above 80%). In this study, 23 male nephropathic patients and 10 age and body composition parameter matched healthy males (control group) were enrolled and took 3 cps/day of OFS for 5 weeks. At the end of the study, the nephropathic patient group showed a statistically significant reduction in the following laboratory parameters: total cholesterol (TC) (*p* = 0.044), atherogenic index TC/high-density lipoprotein cholesterol (*p* = 0.010), inflammatory parameters (C-reactive protein, *p* = 0.048, and erythrocyte sedimentation rate, *p* = 0.019), systolic (*p* = 0.044), and diastolic arterial blood pressure (*p* = 0.003). Regarding body composition, there was an increase in total body water % (*p* = 0.035) with redistribution of extracellular water % (*p* = 0.030) and intracellular water % (*p* = 0.049). In the control group, there was a reduction in fat mass % (*p* = 0.017) and extracellular water % (*p* = 0.047). Therefore, this OFS may represent a valid adjunct therapy to counteract comorbidities related to uremia.

## 1. Introduction

During the last century, a substantial increase in the incidence of chronic noncommunicable diseases (NCDs), such as cardiovascular diseases (CVD), chronic kidney disease (CKD), diabetes mellitus (DM), and cancer, has been observed [1, 2].

Up to this day, NCDs represent the primary cause of death in both developed and developing countries [3]. In this context, prevention, especially through following a healthy diet and leading an active lifestyle, becomes of paramount importance [4, 5]. Amongst NCDs, CKD represents a health problem with significant worldwide impact, its global prevalence being estimated between 7 and 12% [6]. Its increase in prevalence, especially during recent years, is related to different factors. Firstly, it is linked not only to the global ageing of the population [7] but also to the concomitant increase in prevalence of other risk factors such as arterial hypertension (AH), DM, and metabolic syndrome, and thanks to more attentive diagnosis performed by clinicians [8].

Patients affected by CKD frequently present a series of comorbidities, prevalently at the cardiovascular (CV) level; therefore, a new clinical entity has been defined as “type IV cardiorenal syndrome,” characterized by the presence of chronic renal failure which induces a reduction in cardiac function, left ventricular hypertrophy, and increased risk to develop CV complications [9].

Amongst factors related to cardiac dysfunction in CKD patients [10], volume overload and blood pressure increase must be considered as they contribute in producing left ventricular hypertrophy [11].

In fact, CKD patients show an elevated prevalence of AH, principally correlated with extracellular volume expansion [12]; in turn, this induces a decline in cardiac function [13]. A study has highlighted how, in these patients, systolic blood pressure values positively correlate with the expansion of extracellular fluid whilst the latter is inversely correlated with the glomerular filtration rate (GFR) [14].

Other CV risk factors typical of the uremic state are chronic low-grade inflammation, hyperhomocysteinemia [15, 16], insulin resistance, and malnutrition-inflammation-atherosclerosis syndrome [17, 18], which contribute to accelerate the atherosclerotic process. Moreover, the gradual accumulation of uremic toxins in the organism, which increases as GFR decreases, plays a key role in CV alterations [19–21] .

Uremic toxins can precipitate in the progression of CKD, through various mechanisms such as renal fibrosis, loss of antioxidant defenses, dysfunction, and apoptosis of renal tubular cells and endothelial cells, contributing to the generation and propagation of the chronic low-grade inflammatory state which characterizes this pathology [22–24].

Regarding the increment in oxidative stress (OS), which can be observed in CKD patients [25], it is important to consider that the kidney represents one of the most metabolically active organs, which renders it particularly vulnerable to oxidative damage [26–29].

Interestingly, gender differences have been documented in the field of nephrology and in this regard women seem to be protected from developing end-stage renal disease (ESRD) [30, 31].

A screening study has highlighted how the cumulative incidence of ESRD is lower in women during reproductive age and starts increasing 10 years later than in men [31]. This has been confirmed in a Japanese population study, which pointed out that the incidence and the prevalence of ESRD was higher in men compared to women and that the average age at the beginning of renal replacement therapy was higher [30, 31].

In recent years, numerous *in vitro* and *in vivo* studies have focused on researching natural bioactive compounds, which would be ideally free from side effects and would increase the therapeutic potential of standard treatments, as well as having a preventive role in the development of CKD comorbidities [32].

Up to this day, more than 5000 phytocompounds are known, and it is estimated that a large number of these are yet to be discovered [33]. Amongst these, there are vitamins, minerals, flavonoids, phenolic acids, alkaloids, and carotenoids [34–36]. Different classes of phytocompounds act on the organism through various mechanisms and, depending on their polyphenol and antiradical content, perform different antioxidant, cardioprotective, antiproliferative, anti-inflammatory, and hepatoprotective roles.

In particular, the oral food supplement (OFS) used in the present study contains a number of plant dry extracts, listed as follows: *Capsicum annuum* L., which stimulates metabolism; Garcinia cambogia, as a potential antiobesogenic agent [37, 38]; *Centella asiatica* L., which improves microcirculatory parameters [39, 40]; *Cynara scolymus* L. or artichoke, which has an antioxidant and depurative function; and *Aesculus hippocastanum* L. bark extract, which improves the regularity of bowel movements and digestive system functionality.

The present study sets out to evaluate the potential therapeutic effect of this OFS on CV risk and body composition, in male CKD patients versus healthy controls.

## 2. Material and Methods

The study was structured into two phases:

1. *In vitro phase*: qualitative and quantitative HPLC-DAD characterization of the active compounds present in the selected OFS, followed by the evaluation of its antioxidant and free-radical scavenger properties.
2. *In vivo phase*: administration of the characterized OFS to CKD patients and healthy subjects (control group).

### 2.1. Oral Food Supplement, Polyphenol Total Content, and Antioxidant Capacity In Vitro

The OFS used in the present study contains a number of plant dry extracts: *Capsicum annuum* L. present in 60 mg, Garcinia cambogia present in 60 mg, *Centella asiatica* L. present in 100 mg, *Cynara scolymus* L. or artichoke present in 60 mg, and *Aesculus hippocastanum* L. bark extract present in 80 mg. The OFS is formulated in capsules, produced under carefully controlled conditions. Controls are performed continuously throughout the process and guarantee that the capsules conform to the highest quality standards. The excipients used are titanium dioxide (2.0000%) and gelatin (qsp 100%). This OFS has been registered with the Italian Ministry of Health with the number 79086.

The extraction of 400 mg of OFS powder was made in 4.0 ml H_2_O adjusted to pH 2.4 by the addition of HCOOH. The extract was stirred at room temperature for 30 min, centrifuged at 14.000 rpm for 5 min, and analyzed.

#### 2.1.1. HPLC-DAD Analysis

Analyses of flavonols, hydroxycinnamic acids, and coumarins were carried out using an HP 1100 L liquid chromatograph equipped with a DAD detector and managed by an HP 9000 workstation (Agilent Technologies, Palo Alto, CA, USA). Compounds were separated by using a 250 × 4.6 mm i.d. 5 *μ*m LUNA C18 column (Phenomenex, USA). UV/Vis spectra were recorded in the 190-600 nm range, and the chromatograms were acquired at 250, 280, 330, and 350 nm. The samples were analyzed by gradient elution at a flow rate of 0.8 ml/min. The mobile phase was a multistep linear solvent gradient system, starting from 95% H_2_O (adjusted to pH 2 by HCOOH) up to 100% CH_3_CN in 53 minutes. The chemical reagents used were HPLC grade, acetonitrile (CH_3_CN) HPLC grade, ethanol (EtOH) HPLC grade, Folin-Ciocalteu reagent, and sodium carbonate (Na_2_CO_3_); all were purchased from Sigma-Aldrich (St. Louis, Mo, USA).

#### 2.1.2. Identification and Quantification of Individual Compounds

The identity of polyphenols was acquired using data from HPLC-DAD analysis, by comparison with bibliographic data, combination of retention times, and UV/Vis spectra with those of authentic standards. The quantification of individual polyphenolic compounds was performed directly by HPLC-DAD using a five-point regression curve (*R*^2^ ≥ 0.998) in the range of 0-30 *μ*g. In particular, flavonols like the quercetin derivatives were determined at 350 nm using rutin as a reference compound, and hydroxycinnamic acid derivatives were determined at 330 nm using ferulic acid as a reference compound, while coumarins were determined at 330 nm using aesculin as a reference compound. In all cases, actual concentrations of the derivatives were calculated after applying corrections for differences in molecular weight. Each sample was analyzed in triplicate, in order to express the analytical results as an average.

#### 2.1.3. Total Phenolic Content and Total Antioxidant Capacity

The total phenolic content was determined using the Folin-Ciocalteu method, described by Singleton et al. [41] and slightly modified according to Dewanto et al. [42]. To 125 *μ*l of the suitably diluted sample extract, 0.5 ml of deionized water and 125 *μ*l of the Folin-Ciocalteu reagent were added. The mixture was kept in the darkness for 6 minutes, and then 1.25 ml of a 7% aqueous Na_2_CO_3_ solution was added. The final volume was adjusted to 3 ml with water. After 90 minutes, the absorption was measured at 760 nm against water as a blank. The total amount of phenols was expressed as gallic acid equivalents (GAE, mg gallic acid/100 g sample) through the calibration curve of gallic acid. The calibration curve ranged from 20 to 500 *μ*g/ml (*R*^2^ = 0.9969). The phenol content was correlated with the *in vitro* antioxidant activity, as previously confirmed by comparisons with different electron transfer reaction assays and *in vitro* human low-density lipoprotein (LDL) assays [43–45].

#### 2.1.4. Antiradical Activity

Free radical scavenging activity was evaluated with the 1,1-diphenyl-2-picrylhydrazyl radical (DPPH^·^) assay. The antiradical capacity of the sample extracts was estimated according to a slightly modified procedure reported by Brand-Williams et al. [46]. Two ml of the sample solution, suitably diluted with ethanol, was added to 2 ml of an ethanol solution of DPPH^·^ (0.0025 g/100 ml), and the mixture was kept at room temperature. After 20 minutes, the absorption was measured at 517 nm with a Lambda 25 spectrophotometer (PerkinElmer) versus ethanol as a blank. Successively, the absorption of the DPPH^·^ solution was checked. The antiradical activity is calculated by plotting the ratio (*A*_blank_‐*A*_sample_/*A*_blank_) × 100, where *A*_blank_ is the absorption of the DPPH^·^ solution and *A*_sample_ is the absorption of the DPPH^·^ solution after addition of the sample, against the concentration of the sample.

### 2.2. CKD Patients and Control Group

**Figure 1:**
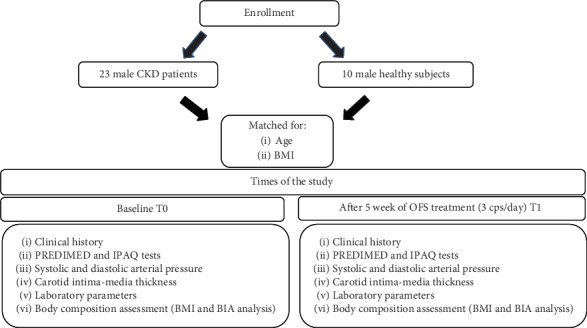
Experimental study design. BMI: body mass index; CKD: chronic kidney disease; IPAQ: International Physical Activity Questionnaire; OFS: oral food supplement; PREDIMED: Prevención con Dieta Mediterránea.

Men affected by CKD and healthy subjects (control group), aged 18-80 years, were considered suitable for the study. The study protocol complied with the declaration of Helsinki and was appointed by the Ethical Committee of Fondazione Policlinico Tor Vergata (PTV) of Rome. The flow chart of the study is summarized in Figure 1.

Men were selected for two fundamental reasons: firstly because of the greater epidemiological toll CKD has on male subjects compared to females and secondly in order to avoid hormonal oscillations typical of women in the reproductive age, which could have influenced measurements regarding body composition.

A fully informed consent form was provided to all subjects prior to the enrolment into the study.

Exclusion criteria were female gender, cancer, hepatitis B and C viruses, human immunodeficiency virus, rheumatologic disorders (e.g., systemic lupus erythematosus), chronic maintenance hemodialysis, body mass index (BMI) < 18.5 kg/m^2^, and consumption of oral supplements and/or vitamins in the last three months.

A total of 23 male patients (mean age 68.5 ± 12.5 years) with CKD (stages I and IV according to the K-DOQI guidelines) [10] were recruited from the Centre of Hypertension and Nephrology Unit of Fondazione PTV of Rome.

In CKD patients, the primary causes of renal failure were chronic glomerulonephritis (17%), nephroangiosclerosis (45%), diabetic nephropathy (9%), autosomal dominant polycystic kidney disease (4%), and other causes (25%).

A complete medical history was recorded for all study participants, in order to gather information about health status, current medications, eating habits, alcohol consumption, smoking, and family history for chronic diseases.

Ten healthy volunteers matched for age and body composition parameters such as weight, height, and BMI, constituted the control group. All enrolled subjects were treated for five weeks with OFS. Each subject consumed a total of 3 cps/day, the first after breakfast, the second after lunch, and the third after dinner, following the recommended dosage for OFS set out by the Italian Ministry of Health, as reported in Section 2.1. The selected daily OFS dose, together with the average dietary polyphenol intake, allows the organism to achieve the optimal daily polyphenol intake requirements in accordance with recent studies [47, 48]. Such intake appears to reduce the insurgence and progression of NCDs and all-cause mortality. To date, this intake value does not have a definitive range; therefore, it is necessary to perform human *in vivo* studies in order to determine the effective daily recommendation dose.

In order to avoid possible biases introduced by lifestyle and dietary regimen modifications, all enrolled subjects were instructed to avoid such changes. To confirm this, the Prevención con Dieta Mediterránea (PREDIMED) questionnaire [49] and the International Physical Activity Questionnaire (IPAQ) [50] were administered at baseline (T0) and after 5 weeks (T1) of OFS supplementation.

Monitoring of laboratory parameters and body composition was conducted at T0 and T1 during the OFS treatment.

### 2.3. Laboratory Parameters

Regarding measurement of laboratory parameters, an automated hematology analyzer XE-2100 (Sysmex, Kobe, Japan) was used for the determination of hemoglobin (Hb). All routine parameters were determined using Dimension Vista 1500 (Siemens Healthcare Diagnostics, Milano, Italy).

The lipid profile, comprised of total cholesterol (TC), triglycerides, LDL cholesterol (LDL-C), and high-density lipoprotein cholesterol (HDL-C), was determined by standard enzymatic colorimetric techniques (Roche Modular P800, Roche Diagnostics, Indianapolis, IN, USA). All other parameters were analyzed according to standard procedures in the Clinical Chemical Laboratories of Fondazione PTV of Rome.

### 2.4. Atherogenic Indices

The atherogenic indices were calculated as described:

- TC (mg/dl)/HDL-C (mg/dl); normal value for male subjects < 5
- LDL-C (mg/dl)/HDL-C (mg/dl); normal value for male subjects < 3.5
- [Log(triglycerides, mg/dl)/HDL-C, mg/dl]; a value < 0.5 is considered as the threshold value above which there is an increased atherogenic risk in both sexes [51, 52].

These indices were determined in order to optimize the predictive capacity of the lipidic profile. In fact, such ratios can supply additional information on CV risk factors which are difficult to quantify with routine laboratory analysis. Moreover, they can depict a more precise picture regarding metabolic and CV alterations, which are closely correlated to lipidic fractions.

### 2.5. Anthropometric Measurements and Body Composition Evaluation

Anthropometric parameters of all the participants were recorded according to standard methods [53]. Body weight (kg) was measured to the nearest 0.01 kg, using a balance scale (seca 711, Hamburg, Germany). Height (m) was measured using a stadiometer to the nearest 0.1 cm (seca 220, Hamburg, Germany). BMI was calculated as body weight divided by height squared (kg/m^2^).

For the evaluation of body composition, all enrolled subjects performed bioelectrical impedance analysis (BIA). Resistance, reactance, impedance, and phase angle at 50 kHz frequency were measured using a BIA 101S instrument (Akern/RIL System-Florence). Body composition analysis was assessed through total body water (TBW), intracellular water (ICW), extracellular water (ECW), body cell mass index (BCMI), fat-free mass (FFM), fat mass (FM), and muscle mass (MM) [54].

### 2.6. Monitoring Systolic and Diastolic Arterial Pressure

Systolic and diastolic blood pressure (BP) was monitored at T0 and T1. Patients were made to sit in a relaxed position for 5 minutes, and then the measurement was taken. The arm cuff was positioned at the level of the heart. BP was measured thrice between intervals of 1-2 minute duration. Ulterior measurements were taken only if the first two presented a difference above 10 mmHg between each other. The reported value was the average between the last two measurements, for both systolic and diastolic BP. During the first visit, BP was measured in both arms; in case of any differences between the two, the highest one was recorded. Additionally, the heart rate was recorded to exclude the presence of eventual arrhythmias [55].

### 2.7. Carotid Intima-Media Thickness Measurement

The carotid intima-media thickness (CIMT) was measured at the beginning and the end of the study. All ultrasound examinations were performed by the same operator (A.N. with 10 years of experience in ultrasound). In our study, a MyLab70 VXG ultrasound device (Esaote, Genova, Italy), with a linear LA523 probe, at a 2–9 MHz frequency range, was used to achieve all examinations. The CIMT was assessed with an ultrasound examination in B-mode, at the level of the right common carotid artery. A longitudinal section of the right common carotid artery was obtained; three different CIMT measurements were performed, about 1 cm below the bifurcation, in the plaque-free area, on the distal wall of the right common carotid artery, using a semiautomatic application. The CIMT thickness was the result of the average value of the three measurements obtained. This procedure was executed in accordance with the Mannheim protocol [56].

### 2.8. Questionnaires

Two questionnaires were administered to the enrolled subjects: PREDIMED and IPAQ at T0 and T1.

The PREDIMED was administered to assess adherence to the Mediterranean diet and in order to make sure that any changes in body composition, inflammatory status, or other laboratory parameters observed in enrolled subjects were really due OFS administration rather than to changes in dietary habits.

The IPAQ was administered to evaluate the degree of physical activity before, during, and after OFS treatment, in order to exclude possible laboratory and body composition changes induced by a different degree of physical activity.

### 2.9. Statistical and Graphical Analysis

Data is reported as means ± standard deviation for parametric variables. All continuous variables were checked for normality using the Kolmogorov-Smirnov test. Differences between the baseline and the final outcomes for parametric values were tested with a paired *t*-test. The minimal level of significance of the differences was fixed at *p* < 0.05. Comparison among groups was performed with the univariate ANOVA with a covariate for continuous parametric variables. Furthermore, the short matrices of data of PREDIMED, IPAQ, and CVD comorbidities were analyzed with McNemar's test [57]. This analysis was performed using the Statistical Package for the Social Sciences Windows, version 15.0 (SPSS, Chicago, Illinois, USA). The graphic result visualization was obtained by GraphPad Prism (La Jolla, CA, USA).

## 3. Results

### 3.1. Chemical Characterization of Food Supplement

**Figure 2:**
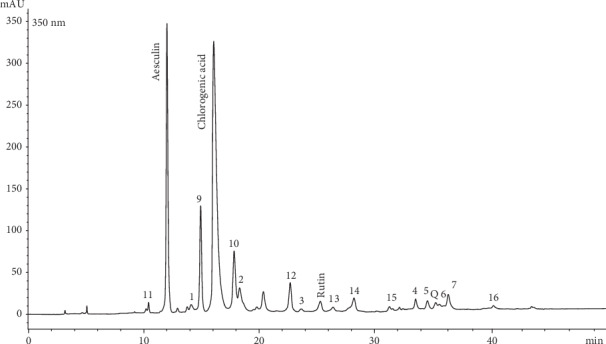
Chromatographic profile acquired at 350 nm of the hydroalcoholic extract. 1-7 = phenolic 435 acids; 9-10 = coumarins; 11-16 = flavonoids (quercetin derivatives); Q = quercetin

Figure 2 shows the chromatographic profile of the OFS extract, acquired at 350 nm. The identified compounds are reported in the legend and include flavonoids, coumarins, phenolic acids, and in particular hydroxycinnamic acids and quercetin derivatives. The HPLC-DAD quantitative analysis shows that the compounds present in major quantities are aesculin and chlorogenic acid.

Table 1 reports the amounts of different subclasses of active compounds present in the OFS analyzed.

Regarding total polyphenol content, antioxidant, and antiradical activity, data was considered from two tests: the Folin-Ciocalteu reagent test, which is related to the antioxidant activity of the commercial product, and the DPPH^·^ test, which accounts for the antiradical activity of the sample. The data is reported in Table 2. The obtained results show that the total content in active antioxidant compounds *per* gram of food supplement is equal to 7.28 GAE expressing a good total antioxidant capacity. Such result can be correlated with the *in vitro* antioxidant capacity on human LDL [43–45]. The total antiradical capacity is above 80% in accordance with a good total antioxidant capacity of the analyzed sample.

### 3.2. Effect of OFS on Body Assessment and Laboratory Parameters in CKD Patients and in Healthy Subjects

The epidemiologic characteristics of the population studied, coupled with a homogeneity evaluation of the two groups relative to age and body composition, are reported in Table 3.

Table 4 summarizes the laboratory parameters examined in patients affected by CKD and in the control group at T0 and T1, with the respective statistical significance relative to the possible effect induced by OFS on such parameters. In particular, a statistically significant reduction in TC at T1 (185.4 ± 47.1 mg/dl vs 176.8 ± 44.7 mg/dl; *p* = 0.044) was observed in CKD patients. Moreover, in nephropathic patients, an improvement in the inflammatory status was also observed at T1, shown by the significant reduction in both C-reactive protein (CRP) (5.1 ± 9.8 mg/dl vs 3.6 ± 7.2 mg/dl; *p* = 0.048) and erythrocyte sedimentation rate (ESR) (48.3 ± 23.2 mm/h vs 40.7 ± 22.6 mm/h; *p* = 0.019).

Regarding the atherogenic indices, as reported in Table 5, at T1, a significant improvement was observed in the ratio between TC and HDL-C (3.8 ± 1.3 vs 3.5 ± 1.2; *p* = 0.010) in CKD patients. Other indices presented no variation secondary to OFS administration. In Table 6, we reported some cardiovascular parameters examined in CKD patients, such as CIMT, by ultrasound examination, and presence of cardiovascular events (like heart attack, stroke, and arrhythmia) at T0 and T1, but these parameters did not show any significant variation in the two time points of the study.

Table 7 shows the parameters relative to the evaluation of body composition, monitored thanks to anthropometric measurements and BIA analysis. In CKD patients, a significant reduction in resistance at T1 (499.4 ± 83.5 ohms vs 486.7 ± 77 ohms; *p* = 0.001) was highlighted. Regarding body water content, a significant increase in TBW was observed at T1 (52.4 ± 6.5% vs 53.3 ± 6.8%; *p* = 0.035) with a better redistribution of water from the extracellular (54.2 ± 6.3% vs 52.7 ± 5.7%; *p* = 0.03) to the intracellular compartment (45.9 ± 6.0% vs 47.2 ± 6.0%; *p* = 0.049). A significant increase in FFM (66.6 ± 8.1% vs 67.6 ± 8.5%; *p* = 0.030) was also noted. The improvement in TBW redistribution has a favorable effect on the systolic and diastolic BP values, as reported in Table 8.

With the aim to exclude the influence on the examined parameters induced by possible lifestyle modification, and in order to evaluate the real effects of the OFS, the PREDIMED and IPAQ questionnaires were administered at T0 and at T1. The PREDIMED evaluated adherence to the Mediterranean Diet, whilst the IPAQ monitored changes in physical activity of enrolled subjects. As highlighted in Tables 9 and 10, in both groups, no lifestyle change was recorded during the study.

Regarding the healthy control group, as shown in Tables 4 and 8, the laboratory parameters do not show any variation after OFS administration. Whereas, for body composition, even in the healthy control group, an increase in TBW (50.5 ± 4.8% vs 51.8 ± 5.4%; *p* = 0.017) associated with a significant reduction in ECW (52.1 ± 4.7% vs 50.7 ± 4.4%; *p* = 0.047) (Table 6) and FM (31.5 ± 6.9% vs 29.8 ± 7.9%; *p* = 0.014) was observed.

## 4. Discussion

This study examined the potential beneficial and cardioprotective effects of an OFS containing natural active compounds from *Capsicum annuum* L., Garcinia cambogia, *Centella asiatica* L., artichoke, and *Aesculus hippocastanum* L. on male nephropathic patients versus healthy subjects. Such OFS was selected on the basis of its anti-inflammatory, antioxidant, and antiradical potential confirmed by qualitative and quantitative chemical characterization of active compounds.

Numerous studies have highlighted that GFR reduction coupled with an increment in albuminuria is associated with CVD [58, 59]. Moreover, the physiological reduction of GFR seems to be correlated with gender differences; in fact, Halbesma et al. [60] underlined gender differences in the annual decrease in GFR, specifically -0.33 ml/min/1.73 m^2^*per* year in women and -0.55 ml/min/1.73 m^2^*per* year in men. The authors have demonstrated that values for systolic BP in women were averagely inferior by 10 mmHg compared to those in men. Considering BP as one of the major determinants of atherosclerosis and progression to ESRD, it can be inferred that gender differences can be partly attributed to this increased systolic BP. Another possible explanation regarding gender difference in GFR decline may be accounted by the different hormonal state observed in the two sexes.

Moreover, it has been estimated, by analyzing urea nitrogen urinary excretion and 24 h sodiuria, that protein and salt intake are lower in female subjects compared to males [60]. Therefore, the well-known CV risk increase in CKD patients is majorly due to the high prevalence of both AH and DM, characteristic of these subjects. AH shows a cause-and-effect relationship with CKD: hypertension represents a risk factor for CKD development, as well as being caused by it [61]. Therefore, in order to prevent CV events in nephropathic patients, the target BP should be lower than 140/90 mmHg, whilst optimal values for a renoprotective effect should be lower than 130/80 mmHg, especially if patients present both decreased GFR and increased albuminuria [62, 63]. This study highlights that after 5 weeks of OFS administration, nephropathic patients experienced a significant reduction in systolic and diastolic BP values. This achievement can be explained by the antihypertensive effect exerted by artichoke, *Centella asiatica* L., and *Capsicum annuum* L. extracts. Precedent *in vitro* studies have demonstrated how artichoke leaf extracts can increase genetic expression of endothelial nitric oxide synthase (eNOS) and induce nitric oxide (NO) production in human vascular endothelial cells [64]. The results are consistent with a randomized placebo-controlled trial conducted on 107 mildly hypertensive or healthy male subjects, which demonstrated a drop in systolic and diastolic blood pressure after 12 weeks oral administration of concentrate artichoke leaf juice [65].

Regarding *Centella asiatica* L., it is hypothesized that its antihypertensive effect is produced by the flavonol quercetin. Such hypothesis was confirmed in an animal study on male Wistar rats, in which hypertension was induced by N-nitro-L-arginine methyl ester (L-NAME) administration. After 30 minutes of L-NAME administration, a *Centella asiatica* L. extract was given, which induced the lowering of systolic and diastolic BP values by acting on eNOS and NO production [66].

An ulterior antihypertensive effect could be correlated to *Capsicum annuum* L. In fact, the active compound present in its extract, named capsaicin, inhibits angiotensin-converting enzyme (ACE) [67] and L-type Ca^2+^ channels present on smooth muscle cells [68]. Moreover, it promotes natriuresis and diuresis, through the stimulation of the transient receptor potential vanilloid type 1 (TRPV1), a receptor involved in hemodynamic and electrolytic homeostasis [69]. Such effect could also explain the reduction in ECW observed in our study in both CKD patients and the control group.

In the healthy control group, the present study highlighted a significant reduction in FM% after OFS administration; such result may be correlated with the antiobesogenic effects of *Capsicum annuum* L., characterized by the activation of thermogenesis, induction of satiety, fat oxidation [70], increase in energy consumption [71], inhibition of adipocyte differentiation [72], and modulation of adipocytokine release [73]. Moreover, FM reduction may also be ascribed to the actions of Garcinia cambogia, such as inducing satiety, body weight maintenance, and stimulation of adipose tissue burning [74]. Consequently, the reduction in FM% determines an increase in TBW%. This is because FFM has a very high water content, estimated at 73% (in multicompartmental models), compared to the relative water paucity which characterizes FM [75].

In this study, TC and atherogenic risk index TC/HDL-C reduction in CKD patients could be attributed to the hypolipidemic effects of artichoke and *Capsicum annuum* L. extracts. In fact, artichoke was proven to act upon lipid metabolism by inducing a reduction in cholesterol production, by reducing its biosynthesis through the inhibition of *β*-hydroxy *β*-methylglutaryl-CoA (HMG-CoA) reductase [76] and by favoring its excretion. Moreover, artichoke increases bile production, which in turn increases cholesterol elimination [77, 78]. Regarding the hypolipidemic action of *Capsicum annuum* L., both *in vitro* and *in vivo* studies have demonstrated how it can reduce TC, triglycerides, and LDL-C, while causing a possible increase in HDL-C [79]. Mueller et al. [80] have demonstrated, in an *in vitro* study, how capsaicin is able to induce a moderate transactivation of a peroxisome proliferator-activated receptor (PPAR*α*), thus positively acting on the lipid profile. This data supports the *in vivo* study by Kwon et al. [81]. The authors subdivided male New Zealand white rabbits into two groups, one was the control group administered a ground-chow diet, containing 1% of cholesterol, and the other was administered the same diet with red pepper addition for 12 weeks. They observed that the red pepper group showed significantly lower activity of cholesteryl ester transfer protein (CETP) and significantly lower levels of TC, triglycerides, and atherogenic index [(TC) − (HDL − C)/(HDL − C)] compared to the control group. The last important effect observed in our study is the improvement in inflammatory parameters in nephropathic patients. Such effect is particularly relevant for clinical implications, because chronic inflammation represents one of the most salient characteristics in the uremic phenotype (correlated with the presence of CVD and protein energy wasting). Moreover, inflammation represents a predictor of negative outcome in nephropathic patients [82–84]. The improvement of the inflammatory state (characterized by the reduction in both CRP and ESR) can be explained by the anti-inflammatory action of *Capsicum annuum* L. A series of studies have showed the anti-inflammatory properties of capsaicin, capable of inhibiting inflammatory mediators such as interleukin-6, tumour necrosis factor-*α*, and E2 prostaglandins. Additionally, an animal study has highlighted how *Capsicum Baccatum* L. juice may exert an anti-inflammatory action by inhibiting cytokine production in proximity of the inflammation site by reducing the recruitment of neutrophils and proinflammatory cytokines in the exudate [85, 86].

During the 9 weeks of OFS administration, we had not observed any CV event (such as heart attack, stroke, or arrhythmia).

In addition, at enrolment, CKD patients showed an increased CIMT with respect to the range of normality [87, 88], suggestive of endothelial dysfunction frequently present in these patients [89]. This parameter did not undergo significant changes at T1, likely due to the short period of OFS administration. However, we observed an improvement in the inflammatory state at T1, which is directly related to CIMT in uremic patients [90]; therefore, it can be hypothesized that by increasing the time of OFS administration, a significant reduction of CIMT can be obtained.

The limitations of the present study reside in the exiguous sample size and the absence of a laboratory parameter monitoring specific oxidative stress biomarkers.

## 5. Conclusions

Our data regarding male nephropathic patients highlighted a significant reduction in systolic and diastolic BP values, TC, and atherogenic index such as TC/HDL-C; moreover, a positive impact on the inflammatory status is observed. All these parameters contribute to the reduction of CV risk in the studied population.

Consequently, the data obtained lays the foundation for the execution of a randomized clinical trial on a larger patient sample, so as to confirm the cardioprotective and anti-inflammatory action exerted by the OFS on nephropathic patients. Moreover, it would be interesting to test the same OFS on patient populations affected by other NCDs like cancer and/or DM.

## Figures and Tables

**Table 1:** Qualitative and quantitative data expressed in mg/g and mg/capsule from HPLC-DAD measurements. The data shown is the mean of three determinations (standard deviation < 3%).

Compounds	mg/g	mg/cps
Phenolic acids	5.49	2.31
Coumarins	1.0	0.42
Flavonoids	0.91	0.38

**Table 2:** Data from the total phenolic content (TPC) expressed as mg GAE/100 g sample; DPPH^·^ data expressed as AA%.

	TPC GAE	AA%
Food supplement sample	7.28	85.17

Abbreviations: TPC: total phenolic content; AA: antiradical activity; GAE: gallic acid equivalents.

**Table 3:** Epidemiological findings of study population and evaluation of the homogeneity of the study groups.

	Case	Control	*p* (ANOVA test)
*N*	23	10	
Age (years)	68.5 ± 12.5^a^	65.1 ± 9.4^a^	ns
Height (m)	1.62 ± 0.09^a^	1.67 ± 0.10^a^	ns
Weight (kg)	75.8 ± 17.3^a^	77 ± 11.2^a^	ns
BMI (kg/m^2^)	28.9 ± 5.3^a^	27.8 ± 4.2^a^	ns

^a^Data expressed as mean ± standard deviation. Abbreviation: BMI: body mass index.

**Table 4:** Laboratory parameters of CKD patients and control group.

	CKD patients			Control group		
	T0	T1	T0 vs T1	T0	T1	T0 vs T1
Creatinine (mg/dl)	2.5 ± 1.4^a^	2.5 ± 1.5^a^	ns^b^	0.8 ± 0.1^a^	0.8 ± 0.1^a^	ns^b^
e-GFR (ml/min/1.72 m^2^)	32.0 ± 18.3^a^	34.1 ± 21.4^a^	ns^b^	93.5 ± 20.4^a^	92.1 ± 14.4^a^	ns^b^
Albuminuria (mg/dl)	424.5 ± 614.5^a^	451.1 ± 651.9^a^	ns^b^	2.5 ± 3.9^a^	12.2 ± 28.1^a^	ns^b^
Azotaemia (mg/dl)	85.0 ± 34.4^a^	80.5 ± 30.8^a^	ns^b^	32.6 ± 8.0^a^	34.8 ± 4.7^a^	ns^b^
Albumin (g/dl)	4.34 ± 0.30^a^	4.20 ± 0.35^a^	ns^b^	4.29 ± 0.2^a^	4.35 ± 0.3^a^	ns^b^
Sodium (mEq/l)	139.78 ± 3.2^a^	140.1 ± 2.6^a^	ns^b^	141.7 ± 2.6^a^	140.0 ± 2.9^a^	ns^b^
Potassium (mEq/l)	4.8 ± 0.6^a^	4.8 ± 0.7^a^	ns^b^	4.5 ± 1.0^a^	4.7 ± 0.6^a^	ns^b^
Calcium (mg/dl)	9.7 ± 0.8^a^	9.7 ± 0.7^a^	ns^b^	9.4 ± 0.4^a^	9.7 ± 0.4^a^	ns^b^
Phosphorus (mg/dl)	3.6 ± 0.8^a^	3.7 ± 0.8^a^	ns^b^	3.5 ± 0.5^a^	3.6 ± 0.5^a^	ns^b^
TC (mg/dl)	185.4 ± 47.1^a^	176.4 ± 44.7^a^	*p* = 0.044^b^	205.8 ± 60.2^a^	214 ± 15.7^a^	ns^b^
HDL-C (mg/dl)	51.5 ± 12.5^a^	52.1 ± 10.9^a^	ns^b^	50.3 ± 13.5^a^	52.9 ± 11.9^a^	ns^b^
Triglycerides (mg/dl)	136.3 ± 59.7^a^	133.8 ± 75.2^a^	ns^b^	100.5 ± 46.2^a^	108.9 ± 66.2^a^	ns^b^
LDL-C (mg/dl)	107.9 ± 39.5^a^	100.9 ± 40.3^a^	ns^b^	127.2 ± 43.2^a^	142.6 ± 12.9^a^	ns^b^
Sideremia (*μ*g/dl)	79.4 ± 29.9^a^	82.0 ± 39.7^a^	ns^b^	89.9 ± 25.5^a^	85.7 ± 20.7^a^	ns^b^
Ferritin (ng/ml)	139 ± 131.2^a^	138.7 ± 129.3^a^	ns^b^	195.2 ± 163.9^a^	179.4 ± 139.7^a^	ns^b^
Transferrin (mg/dl)	247.4 ± 44.5^a^	244.7 ± 40^a^	ns^b^	255.7 ± 39.6^a^	248.5 ± 34.7^a^	ns^b^
Uric acid (mg/dl)	6.0 ± 1.2^a^	6.0 ± 1.4^a^	ns^b^	5.1 ± 1.0^a^	4.9 ± 1.0^a^	ns^b^
CRP (mg/l)	5.1 ± 9.8^a^	3.6 ± 7.2^a^	*p* = 0.048^b^	0.8 ± 0.1^a^	0.8 ± 0.1^a^	ns^b^
ESR (mm/h)	48.3 ± 23.2^a^	40.7 ± 22.6^a^	*p* = 0.019^b^	93.5 ± 20.4^a^	92.1 ± 14.4^a^	ns^b^

^a^Data expressed as mean ± standard deviation; ^b^applied test: *t*-test for paired data. Abbreviations: e-GFR: estimated glomerular filtration rate; TC: total cholesterol; HDL-C: high-density lipoprotein cholesterol; LDL-C: low-density lipoprotein cholesterol; CRP: C-reactive protein; ESR: erythrocyte sedimentation rate. Values of *p* ≤ 0.05 are considered statistically significant.

**Table 5:** Atherogenic indices of CKD patents.

	T0	T1	T0 vs T1
TC/HDL-C	3.8 ± 1.3^a^	3.5 ± 1.2^a^	*p* = 0.010^b^
LDL-C/HDL-C	2.2 ± 2.0^a^	2.1 ± 1.0^a^	ns^b^
Log Trigl/HDL-C	0.044 ± 0.01^a^	0.043 ± 0.01^a^	ns^b^

^a^Data expressed as mean ± standard deviation; ^b^applied test: *t*-test for paired data. Abbreviations: TC: total-cholesterol; LDL-C: low-density lipoprotein cholesterol; HDL-C: high-density lipoprotein cholesterol; Log Trigl: triglyceride logarithm. Values of *p* ≤ 0.05 are considered statistically significant.

**Table 6:** Cardiovascular parameters of CKD patients.

	T0	T1	T0 vs T1
Heart attack (%)	21.7	21.7	ns^b^
Stroke (%)	8.7	8.7	ns^b^
Cardiac arrhythmias (%)	4.3	4.3	ns^b^
Right carotid intima-media thickness (mm)	1.3 ± 0.27^a^	1.2 ± 0.25^a^	ns^c^

^a^Data expressed as mean ± standard deviation; values of *p* ≤ 0.05 are considered statistically significant. Abbreviation: ns: not significant. Applied test: ^b^McNemar's test and ^c^*t*-test for paired data.

**Table 7:** Body composition parameters of CKD patients and control group.

	CKD patients			Control group		
	T0	T1	T0 vs T1	T0	T1	T0 vs T1
Resistance (*R*) (ohm)	499.4 ± 83.5^a^	486.7 ± 77^a^	*p* = 0.001^b^	529.1 ± 56.9^a^	506.9 ± 46.0^a^	ns^b^
Reactance (Xc) (ohm)	40.8 ± 10.9^a^	39.9 ± 11^a^	ns^b^	50.3 ± 9.9^a^	49.1 ± 9.7^a^	ns^b^
Phase angle (°)	4.7 ± 1.1^a^	4.7 ± 1.1^a^	ns^b^	5.4 ± 0.9^a^	5.6 ± 1.0^a^	ns^b^
BMI (kg/m^2^)	28.9 ± 5.3^a^	28.9 ± 5.3^a^	ns^b^	28.9 ± 5.3^a^	28.9 ± 5.3^a^	ns^b^
Weight (kg)	75.3 ± 17.3^a^	76 ± 17.3^a^	ns^b^	75.3 ± 17.3^a^	76 ± 17.3^a^	ns^b^
TBW (%)	52.4 ± 6.5^a^	53.3 ± 6.8^a^	*p* = 0.035^b^	50.5 ± 4.8^a^	51.8 ± 5.4^a^	*p* = 0.017^b^
ICW (%)	45.9 ± 6.0^a^	47.2 ± 6.0^a^	*p* = 0.049^b^	51.1 ± 5.4^a^	51.7 ± 4.8^a^	ns^b^
ECW (%)	54.2 ± 6.3^a^	52.7 ± 5.7^a^	*p* = 0.030^b^	52.1 ± 4.7^a^	50.7 ± 4.4^a^	*p* = 0.047^b^
FM (%)	33.3 ± 7.9^a^	32.4 ± 8.6^a^	ns^b^	31.5 ± 6.9^a^	29.8 ± 7.9^a^	*p* = 0.014^b^
FFM (%)	66.6 ± 8.1^a^	67.6 ± 8.5^a^	*p* = 0.03^b^	68.5 ± 6.9^a^	70.2 ± 8.0^a^	ns^b^
MM (%)	38.8 ± 6.7^a^	39.1 ± 6.0^a^	ns^b^	42.9 ± 6.1^a^	44.6 ± 7.6^a^	ns^b^
BCMI (kg/m^2^)	8.7 ± 1.9^a^	8.8 ± 2.0^a^	ns^b^	9.5 ± 1.4^a^	9.8 ± 1.3^a^	ns^b^

^a^Data expressed as mean ± standard deviation; ^b^applied test: *t*-test for paired data. Abbreviations: BMI: body mass index; TBW: total body water; ICW: intracellular water; ECW: extracellular water; FM: fat mass; FFM: fat-free mass; MM: muscle mass; BCMI: body cellular mass index. Values of *p* ≤ 0.05 are considered statistically significant.

**Table 8:** Blood pressure of CKD patients and control group.

	CKD patients			Control group		
	T0	T1	T0 vs T1	T0	T1	T0 vs T1
Systolic pressure (mmHg)	137 ± 17^a^	131 ± 16^a^	*p* = 0.044^b^	115.5 ± 11.9^a^	110.8 ± 11.2^a^	ns^b^
Diastolic pressure (mmHg)	78 ± 9^a^	75 ± 9^a^	*p* = 0.003^b^	73.1 ± 7.2^a^	72.4 ± 9.1^a^	ns^b^

^a^Data expressed as mean ± standard deviation; ^b^applied test: *t*-test for paired data. Abbreviations: values of *p* ≤ 0.05 are considered statistically significant.

**Table 9:** PREDIMED questionnaire.

	CKD patients			Controls		
	T0	T1	*p* (McNemar's test)	T0	T1	*p* (McNemar's test)
Minimal adherence (%)	8	8	ns	0	0	ns
Average adherence (%)	87	87	ns	80	80	ns
Maximal adherence (%)	5	5	ns	20	20	ns

Abbreviation: ns: not significant.

**Table 10:** IPAQ questionnaire.

	CKD patients			Controls		
	T0	T1	*p* (McNemar's test)	T0	T1	*p* (McNemar's test)
Inactive (%)	57	53	ns	60	60	ns
Sufficiently active (%)	35	39	ns	40	40	ns
Very active (%)	8	8	ns	0	0	ns

## Data Availability

Data are available on request.
